# Evaluation of the Potential Protective Effects of *Lactobacillus* Strains against *Helicobacter pylori* Infection: A Randomized, Double-Blinded, Placebo-Controlled Trial

**DOI:** 10.1155/2022/6432750

**Published:** 2022-09-23

**Authors:** Shumin Wang, Meiyi Zhang, Leilei Yu, Fengwei Tian, Wenwei Lu, Gang Wang, Wei Chen, Jialin Wang, Qixiao Zhai

**Affiliations:** ^1^State Key Laboratory of Food Science and Technology, Jiangnan University, Wuxi, Jiangsu 214122, China; ^2^School of Food Science and Technology, Jiangnan University, Wuxi, Jiangsu 214122, China; ^3^National Engineering Research Center for Functional Food, Jiangnan University, Wuxi, Jiangsu 214122, China; ^4^Department of Emergency, Changhai Hospital, Second Military Medical University, Shanghai 200433, China

## Abstract

**Background:**

The beneficial effects of probiotic supplementation standard antibiotic therapies for *Helicobacter pylori* infection have been verified, but the ability of probiotic monotherapy to eradicate *H. pylori* remains unclear.

**Aim:**

To evaluate the accuracy and efficacy of specific *Lactobacillus* strains against *H. pylori* infection.

**Methods:**

Seventy-eight patients with *H. pylori* infection were treated with strain *L. crispatus* G14-5M (*L. crispatus* CCFM1118) or *L. helveticus* M2-09-R02-S146 (*L. helveticus* CCFM1121) or *L. plantarum* CCFM8610 at a dose of 2 g twice daily for one month. ^14^C-urea breath test, the gastrointestinal symptom rating scale, serum pepsinogen concentrations, and serum cytokine concentrations of patients were measured at baseline and end-of-trial to analyze the effect of the *Lactobacillus* strains in eradicating *H. pylori* infection and reducing gastrointestinal discomfort in patients. In addition, the composition and abundance of the intestinal microbiota of patients were also measured at end-of-trial.

**Results:**

The ^14^C-urea breath test value of the three *Lactobacillus* treatment groups had decreased significantly, and the eradication rate of *H. pylori* had increased by the end of the trial. In particular, the eradication rate in the G14-5M treatment group was significantly higher than the placebo group (70.59% vs. 15.38%, *P*=0.0039), indicating that one-month administration of the G14-5M regimen was sufficient to eradicate *H. pylori* infection. The ingestion of *Lactobacillus* strains also ameliorated the gastrointestinal symptom rating scale scores, and the serum interleukin-8 concentrations of *H. pylori*-infected patients appeared to modulate the gut microbiota of patients. However, none of the *Lactobacillus* strains had a significant effect on general blood physiological characteristics, serum tumor necrosis factor *α* concentrations, or serum pepsinogen concentrations in the patients.

**Conclusion:**

Three *Lactobacillus* strains significantly alleviate the gastrointestinal discomfort and the gastric inflammatory response of *H. pylori*-infected patients. The activity of probiotics in eradicating *H. pylori*infection may be species/strain specific.

## 1. Introduction

*Helicobacter pylori* is a spiral Gram-negative bacterium that colonizes human gastric mucosa [1, 2]. It is associated with diseases of the upper gastrointestinal tract, such as chronic gastritis, peptic ulcers, atrophy of gastric mucosa, mucosa-associated lymphoid tissue lymphoma, and gastric cancer [3, 4]. Standard antibiotic strategies may have adverse consequences, such as causing bacterial antibiotic resistance and gastrointestinal side effects [5, 6]. Thus, several studies have been conducted to develop novel, safe and efficacious therapies to eradicate *H. pylori* in patients. For instance, probiotics improved the eradication rate and reduced side effects when added to the treatments designed to eradicate *H. pylori*. Several food factors proved the antimicrobial activity against *H. pylori*. *β*-caryophyllene, a volatile bicyclic sesquiterpene compound that can be present in the essential oils of many edible plants such as cloves, oregano, and cinnamon, has been reported to significantly inhibit *H. pylori* growth via the downregulation of virulence factors in a model using Mongolian gerbils [7]. The flavonoid compounds baicalin and baicalein found in many medicinal plants exhibit an anti-inflammatory effect. Baicalin and baicalein both suppressed the vacA gene expression of *H. pylori* and interfered with the adhesion and invasion ability of *H. pylori* to human gastric adenocarcinoma cell line (AGS), as well as decreased *H. pylori*-induced interleukin (IL)-8 expression [8]. In the mice infection model, high dosages of baicalin and baicalein inhibited *H. pylori* growth in the mice's stomach [9].

The ability of probiotics to inhibit *H. pylori* infection has been previously demonstrated. In animal models, *Lactobacillus* spp. strongly inhibited *H. pylori* infection by reducing *H. pylori* colonization [10], alleviating *H. pylori*-induced gastric inflammatory responses [11, 12], inhibiting urease activity of *H. pylori* [13], and rebalancing the gastric microbiota [11, 13]. Clinical trials have suggested that a combination of *Lactobacillus* spp. (e.g., *L*. *acidophilus* [14, 15], *L*. *reuteri* [16], *L*. *rhamnosus* [17, 18], *L*. *plantarum* [14], *L*. *bulgaricus* [18], *L*. *casei* [18], and *L*. *sporogenes* [19]) and conventional antibiotic treatment has positive effects on both the eradication rate of *H. pylori* and/or the incidence of overall side effects. A recent meta-analysis (40 articles, 5792 patients) about the efficacy of probiotic-supplemented therapy on the eradication of *H. pylori* and incidence of therapy-associated side effects showed that probiotic supplementation improved the eradication rate by approximately 10% relative to the control group, and the side effects of antibiotic treatment (e.g., diarrhea, vomiting and nausea, constipation, epigastric pain, and taste disturbance) also decreased significantly with probiotic supplementation [20].

The mechanisms by which *Lactobacillus* spp. inhibit *H. pylori* infection are generally as follows [21]: (1) The production of bactericidal metabolites: *Lactobacillus* spp. inhibit *H*. *pylori* growth by producing short-chain fatty acids (e.g., butyrate, propionate, and acetate) and antibacterial agents (e.g., bulgaricus BB18, *L. brevis* BK11, lacticins A164, and lacticins BH5) [12, 22, 23]. For instance, lactacin F, a bacteriocin secreted by *L*. *johnsonii* La1, showed a bactericidal effect against pathogens by forming pores in their lipid bilayers, perturbing membrane permeability and membrane potential [24]. (2) Inhibition of *H. pylori* adherence: *Lactobacillus* spp. affect the adherence of *H*. *pylori* by competing with *H*. *pylori* for attachment to the adhesion receptors for Asialo-GM1 and sulfatide [25], inhibiting expression of the adhesin-encoding gene *sabA* of *H*. *pylori* [26] and upregulating the expression of MUC3mRNA in the gastric mucosa (where MUC3 mucin has the ability to inhibit the adherence of pathogens to epithelial cells) [27], all of which further reduce the *in vivo* colonization of *H*. *pylori*. (3) Modulation of the immune response: *Lactobacillus* spp. decreases the secretion of *H. pylori*-induced IL-8 or tumor necrosis factor (TNF)-*α* and increases the secretion of IL-10 in the gastric mucosa [28, 29].

Although *Lactobacillus* strains used in combination with antibiotics have been shown to eradicate *H. pylori*, few *in vivo* studies have focused on the use of *Lactobacillus* monotherapy to treat *H. pylori* infection. Furthermore, the clinical trial efficacy of single-probiotic strain treatment for *H. pylori* eradication remains controversial. For instance, it was reported that *L. reuteri* treatment (2 × 10^10^ CFU/day) reduced the load of *H*. *pylori* in adults [30], whereas the same dose of *L*. *casei* did not [31]. Similarly, *Lactobacillus* showed strain specificity in the eradication of *H*. *pylori*: *L*. *rhamnosus* GG significantly increased *H*. *pylori* eradication rates in a clinical trial [32], but *L*. *rhamnosus* LR06 had no effect [33].

Thus, there is a clear need for more studies on the effect of treatment with a single-probiotic strain on *H*. *pylori* infection. In our preliminary study, we screened 97 strains of *Lactobacillus* for their ability to inhibit the *in vitro* growth of *H. pylori* (Figure S1), reduce the adherence of *H*. *pylori* to IL-8 cells (Figure S2), and stably colonize C57BL/6 mouse gastric mucosa (Figure S3). We screened out three strains with remarkable bacteriostatic effects, inhibition of *H*. *pylori* adherence, and gastric colonization abilities: *L*. *crispatus* G14-5M, *L*. *helveticus* M2-09-R02-S146, and *L*. *plantarum* CCFM8610. We determined that treatment with each of these *Lactobacillus* strains decreased the concentration of IL-8 secreted by AGS cells cocultured with *H*. *pylori* to a value comparable to the control (Figure S4) and downregulated the expression of the *CagA* gene of *H*. *pylori* (Figure S5). Furthermore, these three strains exhibited the main properties and safety profile required of a probiotic, as follows: resistance to gastrointestinal juices, biliary salts, NaCl, and low pH; the presence of the CRISPR/Cas system (Table S1); no significant toxin-producing virulence factors (Table S2); and low/no harm of antibiotic resistance genes (Table S3 and Figure S6). Therefore, *L*. *crispatus* G14-5M, *L*. *helveticus* M2-09-R02-S146, and *L*. *plantarum* CCFM8610 were selected for a trial in humans.

We aimed to evaluate the accuracy and efficacy of the three *Lactobacillus* strains in eradicating *H*. *pylori* infection in patients, in decreasing their gastrointestinal discomfort, alleviating their gastric inflammatory responses, and regulating their intestinal microbiota.

## 2. Materials and Methods

### 2.1. Patients

The patients were recruited from adults who visited the hospital and had been diagnosed as positive for *H. pylori* infection by a ^13^C/^14^C-urea breath test (UBT), a rapid urease test, or a histological examination of biopsy tissue, within three months before the onset of the study. The exclusion criteria were as follows: the presence of a severe disease, such as malignant tumor and severe metabolic disease; the consumption of nonsteroidal anti-inflammatory drugs, corticosteroids, acid-inhibitory drugs (proton-pump inhibitors or H_2_-receptor blockers), or antiflatulent agents; antibiotic treatment one month prior to study start, including *H*. *pylori* eradication therapy; a habit of ingesting probiotics, yogurt, or lactic acid bacteria-fermented beverages; a history of previous gastrointestinal surgery; mental illness; and pregnancy or lactation.

Seventy-eight individuals were included in the study, and all patients signed a written informed consent form prior to study entrance. The study was conducted at Tinghu District People's Hospital (66 Zhongting Road Middle, Yancheng City, Jiangsu Province, China) from July to November 2019. The clinical trial was approved by the Medical Ethics Committee of Yancheng Tinghu People's Hospital (ET2019033) and was registered in the Chinese Clinical Trial Registry (ChiCTR1900024938).

### 2.2. Experimental *Lactobacillus* Products and the Number of Viable Bacteria

The *Lactobacillus* strains were cultured, lyophilized, and packaged into small aluminum-foil sachets by a probiotic-strain manufacturer (Jiangsu Wecare Biotechnology Co., Ltd., Suzhou, Jiangsu, China). The number of viable bacteria in *Lactobacillus* products during the experimental period was 5 × 10^9^ CFU/g, measured once a week. The placebo products contained soy protein and maltodextrin, provided by the same manufacturer.

All of the products (2 g/sachet) were in a powder form and had the same appearance, packaging, and color. They were stored in a refrigerator at 4°C.

### 2.3. Study Design

The human trial followed a randomized, double-blind, placebo-controlled design. Sample sizes were determined based on similar previous studies [30, 31, 34, 35]. A table of random numbers generated by computer was used to allocate patients to one of four groups, namely, a *L*. *crispatus* G14-5M treatment group (*n* = 19), a *L*. *helveticus* M2-09-R02-S146 treatment group (*n* = 20), a *L. plantarum* CCFM8610 treatment group (*n* = 20) and a placebo group (*n* = 19). Patients were asked to ingest two sachets of probiotic products or placebo products daily (once in the morning and once in the evening) for a month. Both the researchers and the patients were blind to the contents of the products during the study. The patients were followed up weekly by a researcher via phone, who was also unaware of the patient's allocation.

The primary endpoint was a decrease in *H*. *pylori* load evaluated by ^14^C-UBT. The secondary endpoints were a decrease in gastrointestinal discomfort (assessed by a gastrointestinal symptom rating scale (GSRS)), an alleviation of gastric mucosal inflammation (assessed by the ratio of serum pepsinogens [PGs] I and II, and the serum concentrations of inflammatory factors), and changes in the gut microbiota of the patients.

### 2.4. Evaluation Parameters

#### 2.4.1. ^14^C-Urea Breath Test

We used the ^14^C-UBT to confirm the status of *H*. *pylori* infection one day before the treatment and one day after the month-long treatment. Begins with the oral administration of ^14^C labeled urea. *H*. *pylori* produce the urea splitting enzyme Urease, which ultimately cleaves the labeled urea to ammonia and bicarbonate. Bicarbonate is the precursor of CO_2_ that is incorporated into breath. After an overnight fast, all patients swallowed a capsule containing ^14^C-urea with 20 mL of water. Fifteen minutes after capsule intake, each patient blew into a dry cartridge until the breath-card indicator turned from orange to yellow. ^14^CO_2_ collected by the breath card was measured with the *H*. *pylori* analyzer, and disintegrations per minute (DPM) > 100 were judged as positive for *H*. *pylori* infection.

#### 2.4.2. The Gastrointestinal Symptom Rating Scale

The GSRS is a questionnaire recommended by Japanese guidelines for evaluating gastrointestinal symptoms in functional dyspepsia [36].

Each of 15 gastrointestinal symptom items, such as abdominal pain, heartburn, and acid regurgitation, was scored from 0 to 3 according to severity during the past week. A higher score indicated more severe symptoms. The questionnaire was filled in one day before the treatment and one day after the month-long treatment, i.e., a total of two times.

#### 2.4.3. Serum Pepsinogen Concentrations

The blood samples of patients were collected one day before the treatment and one day after the month-long treatment, and serum was obtained by centrifugation. Serum PG (PG I and PG II) concentrations were detected using an enzyme-linked immunosorbent assay (ELISA) kit (Fcmacs Biotech Co., Ltd.), following the protocol recommended by the manufacturer.

#### 2.4.4. Cytokine Analysis

Serum cytokine concentrations were detected using an ELISA kit (Fcmacs Biotech Co., Ltd.), following the protocol recommended by the manufacturer.

#### 2.4.5. Composition and Abundance of the Intestinal Microbiota

Patients provided one stool sample after the completion of the study (within three days). Stool samples were collected in sterile plastic containers and stored at 4°C until they reached the laboratory. Upon arrival, stool samples were immediately stored at −80°C until DNA extraction. DNA was extracted from the stool samples using the FastDNA™ SPIN Kit for Feces (MP Biomedicals, USA), following the manufacturer's protocol. The polymerase chain reaction methods and primers for amplifying the V3-V4 region and the *groEL* gene of the 16S rDNA were based on the previously published protocols [37, 38]. *Lactobacillus*-specific primer sets were developed for the hypervariable region of the groEL gene, a single-copy gene that undergoes rapid mutation and evolution. This methodology could accurately perform taxonomic identification of *Lactobacillus* down to the species level. The accuracy of the method has been demonstrated in fermented yak milk samples and human, rat, and mouse fecal samples.

Library preparation and sequencing were based on the method proposed by Yang et al. [39]. The composition and abundance of the intestinal microbiota of patients were analyzed with the Quantitative Insights Into Microbial Ecology software package (Flagstaff, AZ).

### 2.5. Statistical Analysis

All data were expressed as means ± standard errors of the mean. Fisher's exact tests, one-way analyses of variance (ANOVA), and *t*-tests were performed (using SPSS version 22.0 software) for the comparison of results, such as *H*. *pylori* eradication rate, serum PG concentration, serum cytokine concentration, Shannon index, observed species index and taxa abundance count in different groups. The differences between groups were judged by ANOVA, and the differences between the two groups were judged by a *t*-test or chi-square test. *P* < 0.05 was considered as significant.

## 3. Results

**Figure 1:**
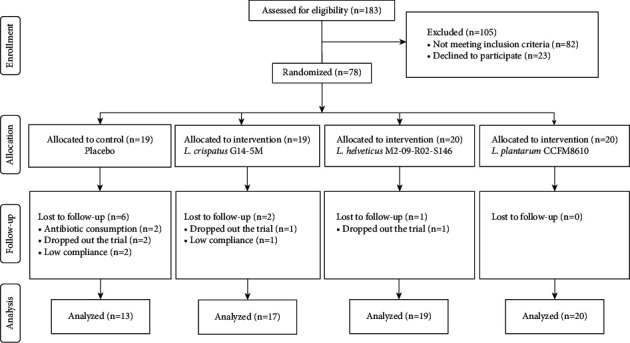
Summary of patient flow in this study.

Seventy-eight patients who were positive for *H. pylori* infection participated in the trial. Six patients in the placebo group, two patients in the G14-5M treatment group, and one patient in the M2-09-R02-S146 treatment group withdrew from the trial, which meant that 69 patients [placebo group (*n* = 13), G14-5M treatment group (*n* = 17), M2-09-R02-S146 treatment group (*n* = 19), and CCFM8610 treatment group (*n* = 20)] completed the study (Figure 1).

### 3.1. General Characteristics of Patients

No statistically significant differences were observed in the mean age, male to female ratio, number of smokers, or number of alcoholic drinkers between the groups of patients who completed the study (Table 1).

Compared with the placebo treatment, the *Lactobacillus* strain treatments did not significantly affect the general blood physiological characteristics of patients (Table 2).

### 3.2. The Eradication Rate of *Helicobacter Pylori*

**Figure 2:**
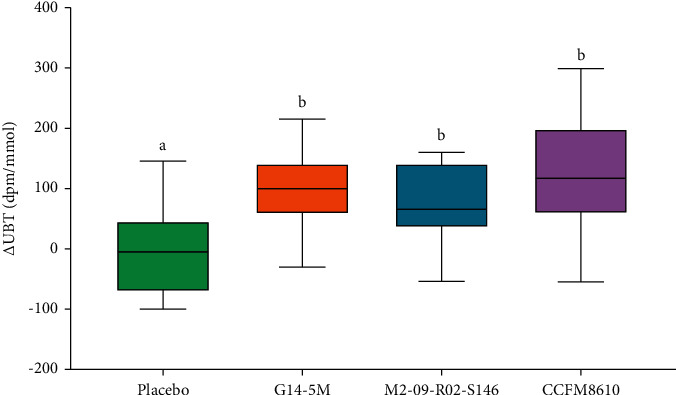
Degree of reduction in ^14^C-urea breath test value.

Compared with the placebo group, the *H. pylori* eradication rate (^14^C-UBT results) was increased in the three *Lactobacillus* treatment groups at the end of the trial, and the eradication rate in the G14-5M treatment group was significantly higher than those of the other groups (Table 3). Specifically, the ^14^C-UBT value of the placebo group showed no significant change before and after the trial, but the ^14^C-UBT values of each of the *Lactobacillus* treatment groups exhibited a significant (70–120 dpm/mmol) decrease (Figure 2).

The letters *a* and *b* above the bars indicate significant differences (*P* < 0.05) between the groups.

### 3.3. Effect of Consumption of *Lactobacillus* Strains on Gastrointestinal Symptom Rating Scale Scores

**Figure 3:**
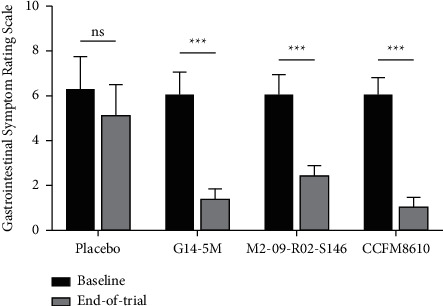
Gastrointestinal symptom rating scale scores of the three treatment groups at baseline and at end-of-trial.

The average GSRS scores of *H*. *pylori*-infected patients in the four groups were all greater than 6.00 at baseline (Figure 3), indicating that they had functional dyspepsia. After one month of treatment with *Lactobacillus* strains, the scores of the three treatment groups were less than 2.50, indicating that their gastrointestinal symptoms were significantly improved compared to baseline (*P* < 0.001).

“ns” indicates no significant differences (*P* > 0.05) between the baseline and end-of-trial.

“^*∗∗∗*^” indicates significant differences (*P* < 0.001) between the baseline and end-of-trial.

### 3.4. Effects of Consumption of *Lactobacillus* Strains on Serum Concentrations of Pepsinogens and Inflammatory Cytokines

Compared with the placebo treatment, the *Lactobacillus* strain treatments did not significantly affect the concentrations of PG I, PG II, or the PG I/PG II ratio in patients' serum (Table 4).

One month after *Lactobacillus* treatment, the mean serum IL-8 concentration in the G14-5M treatment group and the M2-09-R02-S146 treatment group had decreased to 6.16 pg/mL (*P* < 0.05) and 7.09 pg/mL (*P* < 0.01), respectively, which was much lower than the mean serum IL-8 concentration in the placebo treatment group (Table 4). In contrast, treatment with any of the three *Lactobacillus* strains did not cause striking changes in serum TNF-*α* concentrations (*P* > 0.05).

### 3.5. Gut Microbiome Composition in *Helicobacter Pylori*-Infected Patients after *Lactobacillus* Strain Treatment

**Figure 4:**
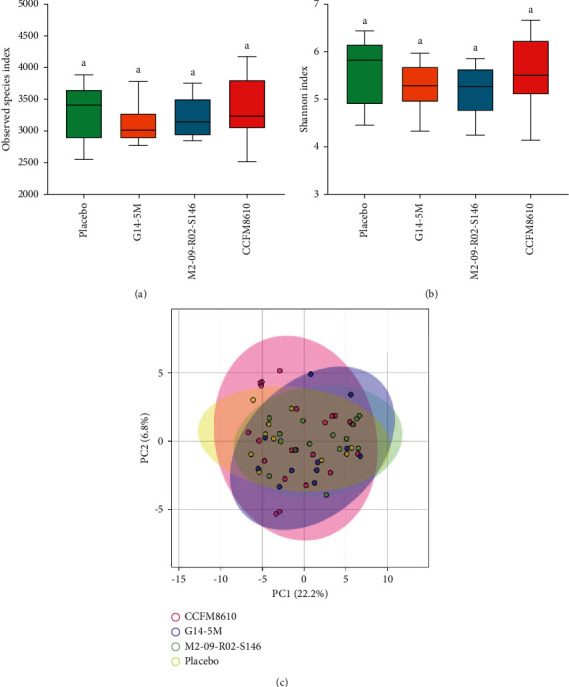
*α*- and *β*-diversity analysis of the gut microbiota at end-of-trial. (a) Observed species index; (b) Shannon index; (c) *β*-diversity, principal component analysis (PCA).

Figures 4(a) and 4(b) indicate that treatment with *Lactobacillus* strains did not affect the richness and diversity of the intestinal microbiota. The result of the *β*-diversity analysis.

Figure 4(c) shows that the distribution of samples in each treatment group was similar and that there was no obvious clustering, indicating that treatment with *Lactobacillus* strains had little effect on the composition and structure of intestinal microbial communities.

The letter *a* above the bars indicates no significant differences (*P* > 0.05) between the groups.

**Figure 5:**
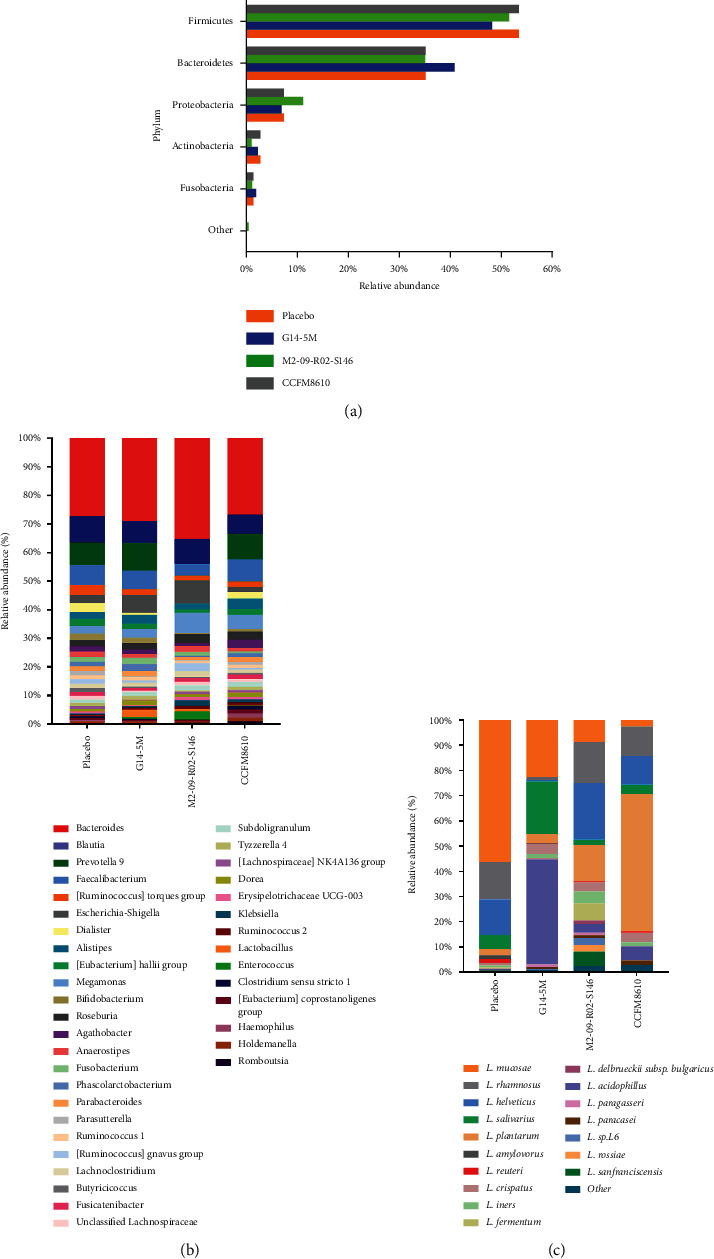
Composition and relative abundance of the gut microbiota at end-of-trial. (a) At the phylum level; (b) at the genus level; (c) at the *Lactobacillus*-species level. The species found at >1% of the average in the total population are shown. Species found at < 1% are grouped as “other”.

Compared with the placebo treatment, the administration of the three *Lactobacillus* strains did not significantly affect the structure of the gut microbiota at the phylum level (Figure 5(a)). Further analysis of the composition at the genus level (Figure 5(b)) showed that all of the treatment groups exhibited an increase in the relative abundances of *Lactobacillus* and *Ruminococcus*, and a decrease in the relative abundances of *Parasutterella* and *Dialister* after one month of *Lactobacillus* strain treatment, relative to placebo. Moreover, compared with the placebo treatment, the relative abundance of *Prevotella* was reduced in the M2-09-R02-S146 treatment group, and the relative abundances of *Escherichia-Shigella* and *Blautia* were reduced in the CCFM8610 treatment group.

There were some differences in the composition of *Lactobacillus* communities at the species level between the four groups (Figure 5(c)). The relative abundances of *L. crispatus*, *L. helveticus,* and *L. plantarum* were increased in the G14-5M, M2-09-R02-S146, and CCFM8610 treatment groups, respectively, consistent with the species of *Lactobacillus* with which each of these groups was treated.

## 4. Discussion

In this double-blind randomized controlled trial, we evaluated the efficacy of *Lactobacillus* strains in eliminating *H. pylori* infection. Compared with the placebo treatment, the ^14^C-UBT value had decreased significantly in the three *Lactobacillus* treatment groups, and the eradication rate of *H. pylori* had increased significantly in the *L. crispatus* G14-5M treatment group at the end of the trial (Figure 2). However, the eradication rates of *H. pylori* in the three *Lactobacillus*-treated groups were different, indicating that the ability of probiotics to inhibit *H. pylori* infection was species-specific, which is consistent with the findings of previous studies [23, 40, 41]. In addition, the types and amounts of short-chain fatty acids and bacteriocins secreted by different *Lactobacillus* species can affect their abilities to inhibit *H*. *pylori* in the stomach [42, 43]. To date, it does not appear clear whether probiotics may be more effective in particular subgroups, and if predictive factors for treatment success can be identified. The complex physiological environment of the human body may affect the ability of probiotics to antagonize *H*. *pylori*. In addition, clinical outcomes may be related to the timing of probiotics intake. Sakamoto et al. [44] reported the efficacy of yogurt containing *L*. *gasseri* OLL2716 (LG21) in suppressing *H*. *pylori*. There was no significant difference in the UBT levels at weeks 0 and 9. However, consumption of the yogurt for 18 weeks reduced gastric mucosal inflammation indicating that long-term administration is necessary. It is also of concern that there are essential factors such as *H*. *pylori* infection strain, the host genetic background, and the host microbiome, that may influence the efficacy of probiotics. Studies indicated that the susceptibility to *H*. *pylori* infection and the outcome of the infection vary according to both *H*. *pylori* and/or host genetic background [45, 46]. In conclusion, further research into the mechanisms underlying the direct and indirect effects of probiotics on *H*. *pylori* could help not only to better refine treatment types but also contribute to a better understanding of some aspects of *H*. *pylori* pathogenesis.

The patients in each group had symptoms of gastrointestinal discomfort before treatment. The *Lactobacillus* treatment groups had significantly lower GSRS scores by the end of the trial, indicating the ability of *Lactobacillus* to relieve gastrointestinal discomfort in patients (Figure 3). Gastrointestinal inflammation and *H*. *pylori* infection may play a role in functional dyspepsia [47]. Several clinical trials have demonstrated that a diet enriched in *Lactobacillus* spp. may alleviate dyspeptic symptoms [34, 41, 48]. The lower incidence of gastrointestinal discomfort in the treatment groups may be due to the suppression of *H*. *pylori* colonization by competition from *Lactobacillus* strains in the gastrointestinal tract. Furthermore, *Lactobacillus* strains may reduce the occurrence of adverse gastrointestinal symptoms by maintaining intestinal homeostasis via creating a lower colonic pH that favors the growth of nonpathogenic species, by stimulating immunity, or by producing antimicrobial substances [49].

IL-8, produced by gastric epithelial cells, is a key cytokine in *H*. *pylori-*associated gastritis [50]. In this study, we demonstrated that the serum IL-8 concentrations of patients in the *Lactobacillus* treatment groups significantly decreased, showing that these treatments had an ameliorative effect on *H. pylori*-related inflammation (Table 4).

Our previous *in vitro* experiments (Figure S4) have also shown that *Lactobacillus* treatment decreased the concentration of IL-8 secreted by AGS cells cocultured with *H*. *pylori*, to a value comparable to the control. Nuclear transcription factor kappa B (NF-*κ*B) is a master regulator of proinflammatory cytokines and antiapoptotic signaling molecules, which can be activated by *H*. *pylori* through several different bacterial components and host signaling pathways [51]. Many investigators have found that specific *Lactobacillus* strains (e.g., *L*. *acidophilus* NCFM and *L*. *salivarius* AR809) inhibit NF-*κ*B signaling pathways, resulting in an attenuation of the secretion of IL-8 [52–54]. In addition, Ryan et al. [55] have proposed that the suppression of IL-8 secretion is a result of *Lactobacillus* spp. downregulating the expression of *CagA* pathogenicity island genes of *H*. *pylori*.

The expression of other proinflammatory cytokines, such as TNF-*α*, increases in *H*. *pylori*-infected mucosa [51]. Serum PG concentrations are associated with the functional activity of the gastric mucosa, and a PGI/PGII ratio < 3 is a marker of atrophic gastritis [56]. In this study, we found that *Lactobacillus* treatment did not affect the serum concentrations of TNF-*α* or PG, which echoes the findings of previous studies [41, 49].

*H*. *pylori* infection elicits significantly different population structures in the gastric, oral and intestinal microbiota, which affects microbiota homeostasis and weakens the body's defense against microorganisms with pathogenic potential [57–59]. Frost et al. [60] identified differences in the relative abundances of 13 intestinal microbiota genera, such as *Bacteroides*, *Prevotella,* and *Parasutterella*, between *H*. *pylori*-infected cases and controls. They also demonstrated that a high abundance of *Prevotella* was positively associated with *H. pylori* infection. In this study, we found that compared with placebo, the *Lactobacillus* strain treatments decreased the relative abundances of *Parasutterella* and *Prevotella* in the intestinal microbiota of patients. The treatments also decreased the abundance of specific gut microbes that have been reported to be associated with oral diseases such as periodontitis (*Dialister*) [61], enteric diseases such as diarrhea (*Escherichia-Shigella*) [62], and metabolic syndromes such as hypertriglyceridemia, fatty liver disease, and insulin resistance (*Blautia*) [63].

Notably, *Lactobacillus* strain treatments also increased the relative abundance of *Ruminococcaceae*, which is an important butyrate-producing family of microbes. Butyrate plays a central role in maintaining gut homeostasis [64, 65]. Furthermore, the colonization of applied *Lactobacillus* strains not only increased the relative abundance of *Lactobacillus* at the genus level but also led to changes in the proportion of various intra-genus species. This may have been due to synergetic or antagonistic interactions between treatment *Lactobacillus* strains and those *Lactobacillus* species that were already present in patients.

*Lactobacillus* strains intervention did not affect the richness and diversity of the intestinal microbiota. Diversity is an important indicator of the productivity, function, and stability of gut microecosystems; however, the diversity in gut microbiota will not be as simple as “more diversity is better” [66]. It is reasonable to conclude that the diversity of the fecal microbiota was not significantly affected by probiotics administration [67]. Probiotics intervention usually significantly altered the proportion of fecal microbiota at the genus level and species level, with the overall community complexity and richness unaffected. This may be due to the influence of intestinal microbiota balance in adults. It may also be attributed to the relatively larger size and the number of overall intestinal microbiota, compared with probiotics administered.

## 5. Conclusion

Overall, the findings demonstrated that the ^14^C-UBT value of the three *Lactobacillus* treatment groups had decreased significantly by the end of the trial. The eradication rate of *H*. *pylori* was significantly elevated by a one-month treatment with a *L*. *crispatus* G14-5M regimen. Treatment with *Lactobacillus* strains also reduced the GSRS score, serum IL-8 concentrations, and the abundance of specific gut microbes that have been linked to *H*. *pylori* infection. The three *Lactobacillus* strains had no significant effect on the physiological indicators of patients. Taken together, these data suggest that the role of probiotics in patients with *H*. *pylori* infection may be species/strain specific.

## Figures and Tables

**Table 1:** General characteristics of patients.

Group	Male/female	Age	Smoking/nonsmoking	Drinking/nondrinking
Placebo (*n* = 13)	2/11	48.15 ± 3.70	0/13	1/12
G14-5M (*n* = 17)	6/11	46.53 ± 2.79	3/14	4/13
M2-09-R02-S146 (*n* = 19)	9/10	54.22 ± 2.70	2/17	3/16
CCFM8610 (*n* = 20)	9/11	48.00 ± 1.74	3/17	5/15
*P*	0.26	0.16	0.47	0.59

**Table 2:** General physiological characteristics of patients.

Parameters	Normal value	Time	Placebo	G14-5M	M2-09-R02-S146	CCFM8610
Red blood cell count (×10^12^/L)	3.50–5.50	Baseline	4.28 ± 0.11	4.59 ± 0.10	4.67 ± 0.10	4.54 ± 0.14
		End-of-trial	4.25 ± 0.15	4.61 ± 0.12	4.60 ± 0.08	4.56 ± 0.12
			*P*=0.89	*P*=0.88	*P*=0.61	*P*=0.93

Platelet (×10^9^/L)	125–320	Baseline	223.64 ± 15.32	215.07 ± 11.79	222.24 ± 10.44	221.90 ± 4.53
		End-of-trial	229.09 ± 15.20	212.21 ± 12.79	228.77 ± 14.04	215.50 ± 11.25
			*P*=0.80	*P*=0.87	*P*=0.71	*P*=0.73

White blood cell count (×10^9^/L)	5–9	Baseline	5.62 ± 0.35	5.62 ± 0.38	5.96 ± 0.29	5.33 ± 0.37
		End-of-trial	6.34 ± 0.47	5.41 ± 0.37	6.31 ± 0.37	5.46 ± 0.29
			*P*=0.23	*P*=0.70	*P*=0.46	*P*=0.79

Hemoglobin (g/L)	120–185	Baseline	126.55 ± 3.17	134.93 ± 4.71	142.94 ± 3.65	136.30 ± 4.53
		End-of-trial	126.09 ± 4.96	136.93 ± 5.17	143.29 ± 3.14	136.80 ± 4.40
			*P*=0.94	*P*=0.78	*P*=0.94	*P*=0.94

Fasting blood sugar (mmoL/L)	3.9–6.1	Baseline	5.38 ± 0.20	5.19 ± 0.21	5.24 ± 0.24	5.23 ± 0.16
		End-of-trial	5.74 ± 0.32	5.11 ± 0.21	5.84 ± 0.58	5.53 ± 0.40
			*P*=0.36	*P*=0.80	*P*=0.34	*P*=0.46

Glutamic-pyruvic transaminase (U/L)	0–40	Baseline	18.55 ± 2.71	14.64 ± 1.43	30.64 ± 5.64	20.10 ± 2.37
		End-of-trial	26.45 ± 6.91	18.64 ± 2.11	34.29 ± 6.33	23.75 ± 2.79
			*P*=0.30	*P*=0.13	*P*=0.67	*P*=0.33

Total bilirubin (*μ*mol/L)	5.13–22.24	Baseline	12.93 ± 1.97	12.39 ± 1.49	13.85 ± 1.13	14.64 ± 0.98
		End-of-trial	12.86 ± 1.65	13.19 ± 1.85	13.31 ± 0.98	14.71 ± 1.45
			*P*=0.98	*P*=0.74	*P*=0.72	*P*=0.97

Glutamic-oxaloacetic transaminase (U/L)	0–40	Baseline	19.64 ± 1.19	17.64 ± 0.89	25.12 ± 2.34	20.50 ± 0.94
		End-of-trial	23.00 ± 3.90	17.71 ± 1.13	22.76 ± 2.07	21.55 ± 1.51
			*P*=0.42	*P*=0.96	*P*=0.46	*P*=0.56

Alkaline phosphatase (U/L)	45–135	Baseline	78.91 ± 5.67	65.86 ± 6.08	73.82 ± 5.91	69.05 ± 6.70
		End-of-trial	72.82 ± 5.78	67.21 ± 6.02	74.88 ± 6.01	72.40 ± 7.07
			*P*=0.46	*P*=0.88	*P*=0.90	*P*=0.73

**Table 3:** *Helicobacter pylori* infection-eradication rate.

Analysis set	Group	Negative (*n*)	Positive (*n*)	Eradication rate (%)
PP	Placebo (*n* = 13)	2	11	15.38
	G14-5M (*n* = 17)	12	5	70.59^*∗∗*^
	M2-09-R02-S146 (*n* = 19)	10	9	52.63
	CCFM8610 (*n* = 20)	9	11	45.00

ITT	Placebo (*n* = 17)	4	13	23.53
	G14-5M (*n* = 18)	12	6	66.67^*∗*^
	M2-09-R02-S146 (*n* = 19)	10	9	52.63
	CCFM8610 (*n* = 20)	9	11	45.00

“^*∗∗*^” (*P*=0.0039) and “^*∗*^” (*P*=0.0176) indicate significant differences between the G14-5M treatment group and the placebo group. PP: per-protocol analysis; ITT: intention-to-treat population. The data of the placebo group have been previously published in *Food and Fermentation Industries* (DOI: 10.13995/j.cnki.11-1802/ts.024742).

**Table 4:** Effects of *Lactobacillus* strain consumption on serum concentrations of pepsinogens and inflammatory cytokines.

Parameters	Group	Baseline	End-of-trial	*P*
PG I (ng/mL)	Placebo	107.61 ± 14.47	104.07 ± 11.01	0.85
	G14-5M	83.78 ± 5.80	89.66 ± 6.10	0.49
	M2-09-R02-S146	114.98 ± 10.43	113.90 ± 8.51	0.94
	CCFM8610	101.09 ± 11.37	102.48 ± 7.44	0.92

PG II (ng/mL)	Placebo	18.03 ± 2.77	15.75 ± 2.11	0.52
	G14-5M	16.42 ± 2.09	13.82 ± 1.88	0.35
	M2-09-R02-S146	19.42 ± 1.85	15.08 ± 1.57	0.08
	CCFM8610	18.43 ± 1.55	14.68 ± 1.10	0.06

PG I/PG II	Placebo	6.60 ± 0.64	7.74 ± 1.06	0.37
	G14-5M	5.82 ± 0.86	7.79 ± 1.06	0.16
	M2-09-R02-S146	6.50 ± 0.67	8.50 ± 1.03	0.12
	CCFM8610	6.01 ± 0.70	7.56 ± 0.71	0.13

IL-8 (pg/mL)	Placebo	11.41 ± 0.98	7.40 ± 1.78	0.08
	G14-5M	10.96 ± 1.42	6.16 ± 1.76	0.049^*∗*^
	M2-09-R02-S146	13.60 ± 1.35	7.09 ± 1.74	0.008^*∗∗*^
	CCFM8610	12.12 ± 1.11	8.67 ± 2.47	0.20
	Placebo	13.00 ± 0.35	12.11 ± 0.37	0.09
	G14-5M	12.72 ± 0.27	12.64 ± 0.91	0.93

TNF-*α* (pg/mL)	M2-09-R02-S146	13.64 ± 0.61	12.81 ± 0.61	0.34
	CCFM8610	13.46 ± 0.66	12.98 ± 0.74	0.63

“^*∗*^” indicates significant differences (*P* < 0.05) between baseline and end-of-trial. “^*∗∗*^” indicates significant differences (*P* < 0.01) between baseline and end-of-trial.

## Data Availability

The data used to support the findings of this study are included within the article and the supplementary materials.

## Supplementary Materials

Supplementary Materials Table S1. distribution of the CRISPR-Cas regions among *Lactobacillus* strains. Table S2. potential virulence factor of *Lactobacillus* strains. Table S3.distribution of the intact prophage regions among *Lactobacillus* strains. Table S4. antibiotic resistance of *Lactobacillus* strains. Figure S1. the inhibition of *Lactobacillus* strains on the growth of *Helicobacter pylori*. Figure S2. inhibitory effect of *Lactobacillus* strains on the adhesion of *H. pylori* to the human gastric adenocarcinoma cell line (AGS). Figure S3. colonization of *Lactobacillus* strains in the stomach of mice. Figure S4. changes in IL-8 production of AGS after *Lactobacillus* strains intervention. Figure S5. changes in virulence factor expression after *Lactobacillus* strains intervention. Figure S6. distribution of resistance genes in *Lactobacillus* strains.
