# Supplementary material for: Evaluation of the Potential Protective Effects of Lactobacillus Strains against Helicobacter pylori Infection: A Randomized, Double-Blinded, Placebo-Controlled Trial
Source: Can J Infect Dis Med Microbiol. 2022 Sep 23;2022:6432750. doi: 10.1155/2022/6432750 (PMC9525740; doi:10.1155/2022/6432750)
Supplement: Supplementary Materials — Table S1. distribution of the CRISPR-Cas regions among Lactobacillus strains. Table S2. potential virulence factor of Lactobacillus strains. Table S3.distribution of the intact prophage regions among Lactobacillus strains. Table S4. antibiotic resistance of Lactobacillus strains. Figure S1. the inhibition of Lactobacillus strains on the growth of Helicobacter pylori. Figure S2. inhibitory effect of Lactobacillus strains on the adhesion of H. pylori to the human gastric adenocarcinoma cell line (AGS). Figure S3. colonization of Lactobacillus strains in the stomach of mice. Figure S4. changes in IL-8 production of AGS after Lactobacillus strains intervention. Figure S5. changes in virulence factor expression after Lactobacillus strains intervention. Figure S6. distribution of resistance genes in Lactobacillus strains. [file 6432750.f1.docx]

**Supplementary Materials**

The more detailed screening methods for the three *Lactobacillus* strains used in this trial are provided as supplementary material, including the results of *in vitro* cellular experiments, *in vivo* animal experiments and genomic analysis of the strains. The data of supplementary materials has not been published before.


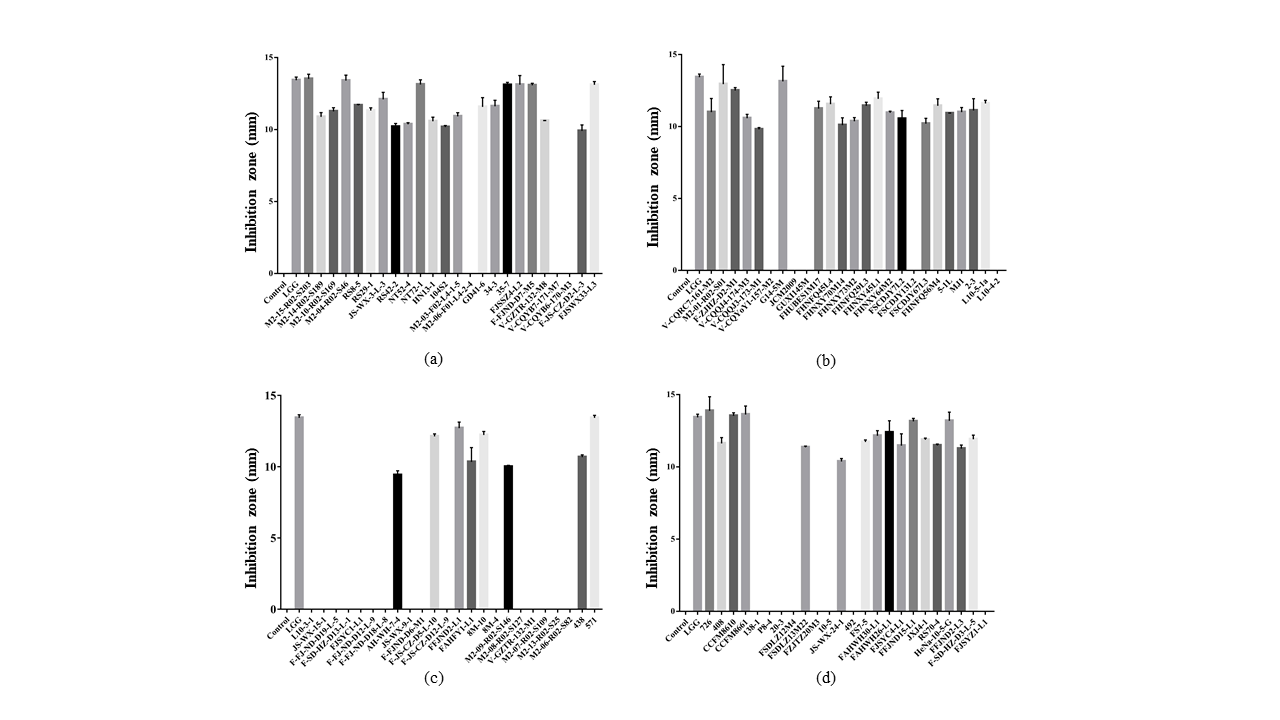
In our preliminary study, we determined the inhibition of *H. pylori* growth by 97 strains of *Lactobacillus*, comprising 11 *Lactobacillus* spp. (Figure S1). In addition to the negative control (MRS medium at pH 6.2), 32 *Lactobacillus* strains showed no inhibition, the positive control *Lactobacillus* *rhamnosus* GG had an inhibition zone diameter of 13.43 mm, the other 65 *Lactobacillus* strains had inhibition zones ranging from 9.41 mm to 13.88 mm in diameter, with differences in inhibition capacity.

Figure S1: The inhibition of *Lactobacillus* strains on the growth of *Helicobacter pylori*.


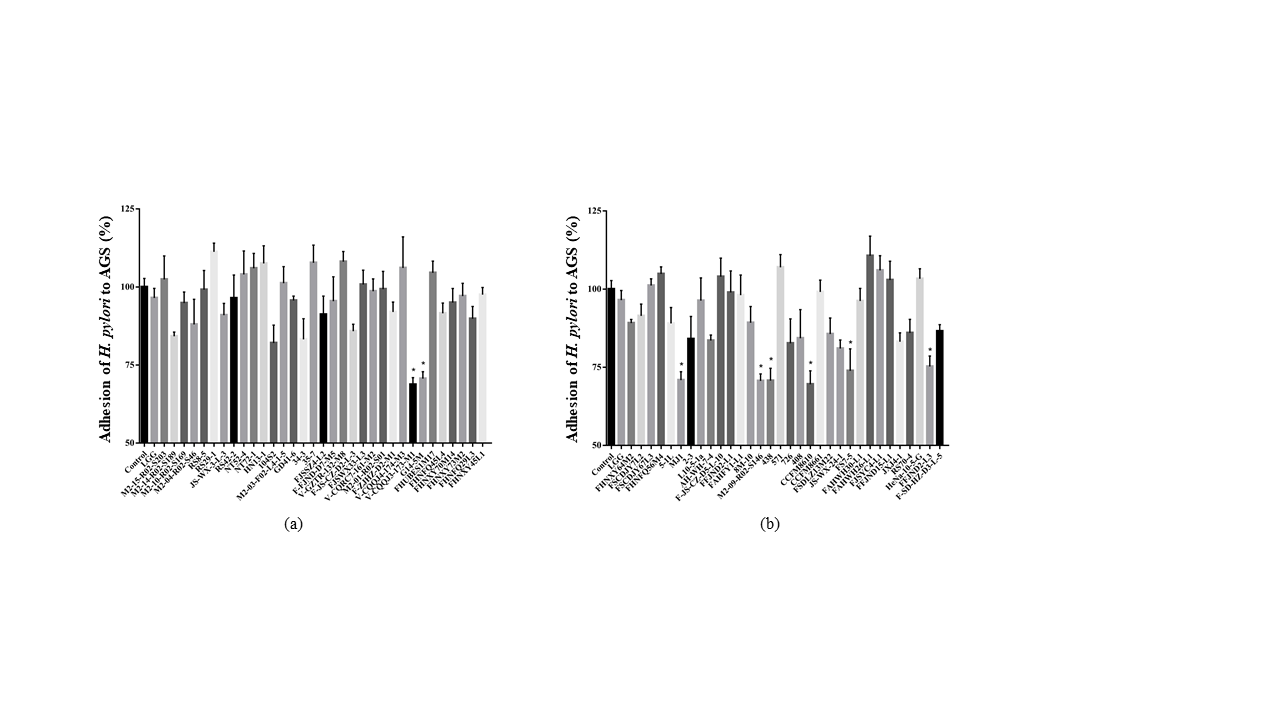
We further tested the inhibitory effect of these 65 *Lactobacillus* strains on the adhesion of *H. pylori* to AGS (Figure S2). The ability of different *Lactobacillus* strains to inhibit the adhesion of *H. pylori* varied greatly, only 8 of the 65 *Lactobacillus* strains significantly reduced the adhesion rate of *H. pylori*. After treatment with these 8 *Lactobacillus* strains, the adhesion rate of *H. pylori* was reduced to less than 75%.

Figure S2: Inhibitory effect of *Lactobacillus* strains on the adhesion of *H. pylori* to the human gastric adenocarcinoma cell line (AGS).

“*” indicates significant differences (*P* < 0.05) between the treatment groups and the placebo group.

A total of 6 *Lactobacillus* strains were selected *in vitro* based on their ability to inhibit *H. pylori* growth and to reduce *H. pylori* adhesion to AGS. They were *L. casei* V-CQQJ3-173-M1, *L. crispatus* G14-5M, *L. fermentum* MJ1, *L. helveticus* M2-09-R02-S146, *L. plantarum* CCFM8610 and *L. salivarius* FFJND2-L3. These strains were used for the next *in vivo* screening test, based on their ability to colonize the stomach of mice. We measured the colonization of *Lactobacillus* strains in stomachs of mice after one or seven times of gavage of the *Lactobacillus* strain.

As shown in the Figure S3, the results revealed that the trends of the reduction in colonization level and abundance of *Lactobacillus* were similar in both methods. After gavage of *L. casei* V-CQQJ3-173-M1, it did not colonize in mice, the abundance of V-CQQJ3-173-M1 in the stomach was always below the detection limit of the qPCR standard curve. The detection levels of the remaining five *Lactobacillus* strains in the stomach of mice after gavage were significantly higher than the abundance detected in the control group. The initial levels of five species of *Lactobacillus* in the stomach of mice were low (10-10^2^ CFU), however, after gavage of *Lactobacillus* strains, the levels of *Lactobacillus* strains were significantly increased (*P* < 0.05). The detection levels of *L. crispatus* G14-5M, *L. helveticus* M2-09-R02-S146 and *L. plantarum* CCFM8610 were still significantly higher than the control group after 3 days of discontinuation of gavage (*P* < 0.05), while the abundance of *L. fermentum* MJ1 and *L. salivarius* FFJND2-L3 decreased to the initial level on the second day of discontinuation of gavage.


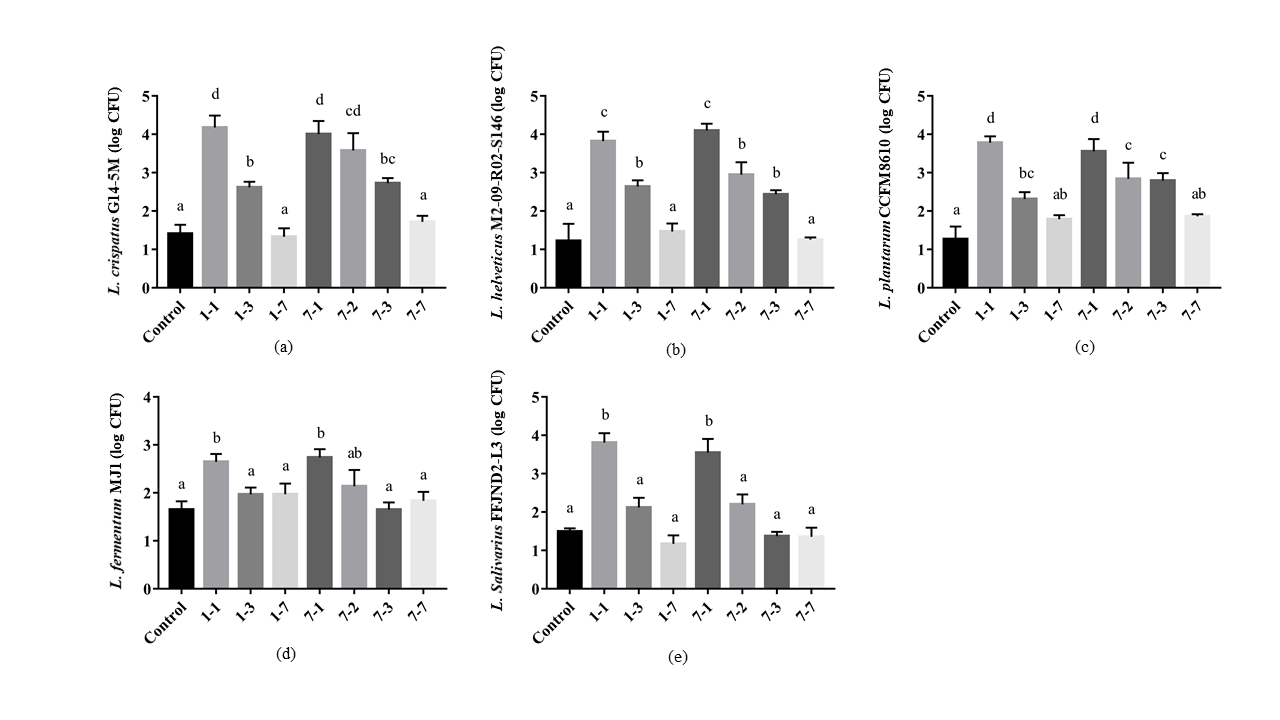
Figure S3: Colonization of *Lactobacillus* strains in the stomach of mice.

Different letters “a, b, c and d” indicate significant differences (P < 0.05) between groups.

Over all, by evaluating the colonization characteristics of *Lactobacillus* stains in the stomach of mice, three strains (*L. crispatus* G14-5M, *L. helveticus* M2-09-R02-S146 and *L. plantarum* CCFM8610) were selected for the next stage of the study.


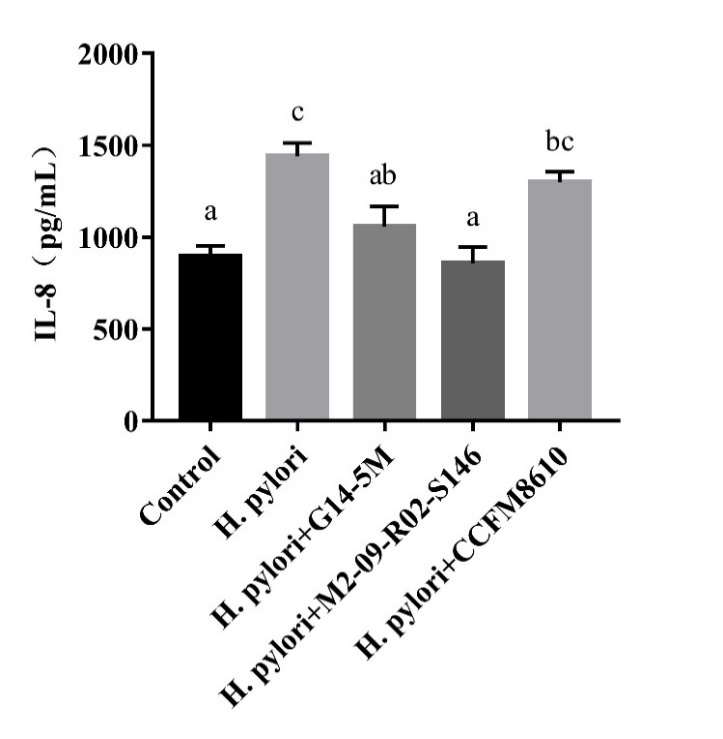
The levels of IL-8 secreted by the AGS increased to 1500 pg/mL after *H. pylori* infection, which was significantly higher than the level of uninfected controls (*P* < 0.05). After *L. crispatus* G14-5M and *L. helveticus* M2-09-R02-S146 interventions, the levels of IL-8 decreased to the level comparable to that of uninfected control with *H. pylori* infection (Figure S4).

Figure S4: Changes in IL-8 production of AGS after *Lactobacillus* strains intervention.

Different letters “a, b and c” indicate significant differences (*P* < 0.05) between groups.


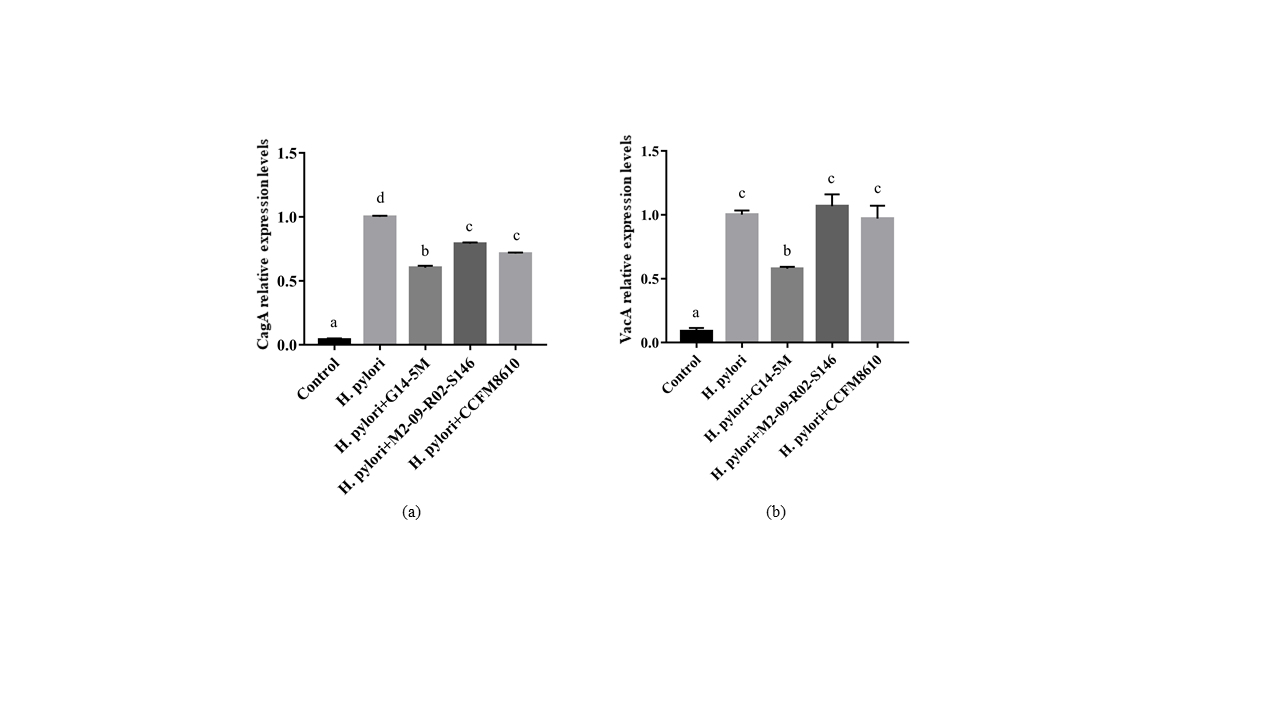
Two virulence factors, *CagA* and *VacA*, play a major role in the pathogenesis of *H. pylori*. As shown in the Figure S5, both virulence factors were barely expressed in the non-*H. pylori* infected AGS (the control group) compared to the *H. pylori* infected group, while both virulence factors were down-regulated to different degrees after *Lactobacillus* strains intervention. *L. helveticus* M2-09-R02-S146 and *L. plantarum* CCFM8610 down-regulated the expression of *CagA* gene, while the down-regulation of *VacA* gene expression was not significant. In contrast, *L. crispatus* G14-5M significantly down-regulated both *CagA* and *VacA* gene expression.

Figure S5: Changes of virulence factor expression after *Lactobacillus* strains intervention.

Different letters “a, b and c” indicate significant differences (P < 0.05) between groups.

The CRISPR-Cas system was predicted for these three strains, and the presence or absence of the CRISPR-Cas system in the strains was determined by the presence or absence of CRISPR repeats greater than 4 and the presence or absence of the Cas gene.

As shown in the Table S1, all three *Lactobacillus* strains had CRISPR-Cas systems, among which *L. crispatus* G14-5M had two CRISPR-Cas systems and the Cas gene was located upstream of the CRISPR sequence, indicating that the CRISPR-Cas systems of these three strains were active and could resist the invasion of exogenous DNA. Due to the difference of Cas gene, the CRISPR-Cas systems of these three strains were divided into different types, i.e., the three strains had different mechanisms to degrade exogenous DNA. Among them, *L. crispatus* G14-5M and *L. plantarum* CCFM8610, both of which have type IIA CRISPR-Cas systems, are consistent with previous reports.

Table S1: Distribution of the CRISPR-Cas regions among *Lactobacillus* strains

| ***Lactobacillus* strain** | **CRISPR-Cas type** | **CRISPR repeat sequence area** | **CRISPRspacer** | **Cas type** |
| --- | --- | --- | --- | --- |
| G14-5M | TypeⅠB | GTATTTATTTAACTTAAGAGGAATGTAAAT | 15 | Cas6，Cas8，Cas7，Cas5，Cas3，Cas4，Cas1，Cas2，Cas3 |
|  | TypeⅠE | GTATTCTCCACGCATGTGGAGGTGATCC | 15 | Cse2，Cas7，Cas5，Cas6，Cas1，Cas2 |
| M2-09-R02-S146 | TypeⅠC | GTCGCACTCCTTGTGAGTGCGTGGATTGAAAT | 18 | Cas1，Cas5，Cas7，Cas8 |
| CCFM8610 | TypeⅡA | GTCTTGAATAGTAGTCATATCAAACAGGTTTAGAAC | 13 | Cas9，Cas1，Cas2，Csn2 |

The pathogenicity of bacteria is often closely related to the presence of virulence factors. In this study, the genes of the *Lactobacillus* strains were compared with the database of virulence genes (Table S2). 25, 24 and 28 homologous sequences of known virulence factors were found in *L. crispatus* G14-5M, *L. helveticus* M2-09-R02-S146 and *L. plantarum* CCFM8610, respectively. The majority of these genes are mainly involved in the degradation of substances such as carbohydrates (e.g. LOS), the transport of metal ions (e.g. HitABC) and the synthesis of extracellular structures such as capsular polysaccharide (e.g. Capsule). These genes have not been reported to be associated with the pathogenic features of *Lactobacillus*. The remaining genes are mainly related to bacterial adhesion properties (e.g., Hsp60), which can assist in the adhesion and colonization of *Lactobacillus* in the gastrointestinal tract. In a word, no significant toxin-producing virulence factors were found in these three *Lactobacillus* strains, and the strains were not significantly pathogenic.

Table S2: Potential virulence factor of *Lactobacillus* strains

| **VFDB ID** | **Virulence factors** | **Function** | **Initial source** |
| --- | --- | --- | --- |
| VFG002189 | Capsule | undecaprenyl diphosphate synthase | *E.faecalis* |
| VFG002181 | CpsJ | ABC transporter, ATP-binding protein | *E.faecalis* |
| VFG002190 | CpsA | undecaprenyl diphosphate synthase | *E.faecalis* |
| VFG002182 | CpsI | UDP-galactopyranose mutase | *E.faecalis* |
| VFG045681 | CdsA | phosphatidate cytidylyltransferase | *E.faecalis* |
| VFG001322 | Isd | iron-regulated surface determinant protein F, ATP-binding-cassette-type transmembrane transporter | *S.aureus* |
| VFG000344 | HitABC | iron (III) ABC transporter, ATP-binding protein | *H.influenzae* |
| VFG001206 | FbpABC | iron (III) ABC transporter, ATP-binding protein | *N.meningitidis* |
| VFG045566 | Dot/Icm | Dot/Icm type IV secretion system effector | *L.pneumophila* |
| VFG006717 | Lap | Listeria adhesion protein Lap | *L.monocytogenes* |
| VFG045346 | IlpA | immunogenic lipoprotein A | *V.vulnificus* |
| VFG042979 | Ebp pili | sortase | *E. faecalis* |
| VFG001329 | PavA | adherence and virulence protein A | *S.agalactiae* |
| VFG001983 | PEB1 | bifunctional adhesin/ABC transporter  aspartate/glutamate-binding protein | *C.jejuni* |
| VFG000964 | Hyaluronic  acid capsule | UDP-glucose pyrophosphorylase | *S.pyogenes* |
| VFG001855 | Hsp60 | Hsp60, 60K heat shock protein HtpB | *L.pneumophila* |
| VFG037100 | MsrAB | trifunctional thioredoxin/methionine sulfoxide  reductase A/B protein | *N.meningitidis* |
| VFG037046 | MntABC | ABC transporter ATP-binding protein MntA | *N.meningitidis* |
| VFG000077 | ClpP | ATP-dependent Clp protease proteolytic subunit | *L.monocytogenes* |
| VFG000079 | ClpC | endopeptidase Clp ATP-binding chain C | *L.monocytogenes* |
| VFG037118 | RecN | DNA repair protein RecN | *N.meningitidis* |
| VFG000080 | ClpE | ATP-dependent protease | *L.monocytogenes* |
| VFG000077 | Clp | ATP-dependent Clp protease proteolytic subunit | *L.monocytogenes* |
| VFG001386 | PhoP | Possible two component system response  transcriptional positive regulator PhoP | *M.tuberculosis* |
| VFG001826 | RelA | Probable GTP pyrophosphokinase RelA | *M.tuberculosis* |
| VFG001269 | Cya | cyclolysin secretion ATP-binding protein | *B.pertussis* |
| VFG002161 | Lsp | signal peptidase II | *L.monocytogenes* |
| VFG002197 | BopD | sugar-binding transcriptional regulator, LacI family | *E.faecalis* |
| VFG032992 | OatA | peptidoglycan O-acetyltransferase | *L.monocytogenes* |
| VFG012509 | Salmochelin | ATP binding cassette transporter | *E.coli* |
| VFG032878 | PrsA2 | post translocation chaperone PrsA2 | *L.monocytogenes* |
| VFG045470 | Cytolysin | cytolysin regulator R2 | *E.faecalis* |
| VFG000841 | Hemolysin | hemolysin transport protein | *E.coli* |
| VFG005767 | Beta-hemolysin/  cytolysin | 3-ketoacyl-ACP-reductase CylG | *S.agalactiae* |
| VFG002158 | LplA1 | lipoate protein ligase | *L.monocytogenes* |
| VFG002050 | T2SS | general secretion pathway protein G | *S.dysenteriae* |
| VFG000670 | LPS | bactoprenol glucosyl transferase | *S.flexneri* |
| VFG013327 | LOS | phosphomannomutase | *H.influenzae* |
| VFG030686 | SugC | probable sugar ABC transporter, ATP-binding  protein SugC | *M. abscessus* |
| VFG006809 | Lgt | prolipoprotein diacylglyceryl transferase | *L. welshimeri* |
| VFG002165 | EfaA | endocarditis specific antigen | *E. faecalis* |
| VFG006062 | RmlB | dTDP-glucose-4,6-dehydratase | *S. thermophilu* |
| VFG006031 | EpsE | exopolysaccharide biosynthesis protein,  glycosyl-1-phosphate transferase | *S. thermophilus* |
| VFG016532 | OppF | oligopeptide ABC transporter, permease  component | *M. mycoides* |
| VFG004585 | SdrF | Ser-Asp rich fibrinogen-binding, bone  sialoprotein-binding protein | *S. epidermidis* |
| VFG026754 | NarG | Putative respiratory nitrate reductase | *M. canettii* |
| VFG032626 | LisR | putative two-component response regulator | *L. ivanovii* |
| VFG009361 | TrpD | anthranilate phosphoribosyltransferase | *M. smegmatis* |
| VFG032819 | DltA | putative D-alanine-activating enzyme | *L. ivanovii* |
| VFG031404 | CtpV | metal cation transporter p-type ATPase, CtpV | *M. intracellulare* |
| VFG000925 | FepC | ferrienterobactin ABC transporter ATPase | *E. coli* |
| VFG000925 | Enterobactin | ferrienterobactin ABC transporter ATPase | *E.coli* |
| VFG002162 | BSH | bile salt hydrolase | *L.monocytogenes* |
| VFG000574 | MgtBC | Mg2+ transport protein | *S.enterica* |

We predicted the resistance genes of three *Lactobacillus* strains (Figure S6). It shown that *L. crispatus* G14-5M, *L. helveticus* M2-09-R02-S146 and *L. plantarum* CCFM8610 carried 19, 16, and 55 resistance genes, respectively, among which *L. plantarum* CCFM8610 carried relatively more resistance genes. These three strains mainly carried resistance genes for five classes of antibiotics: aminoglycosides,
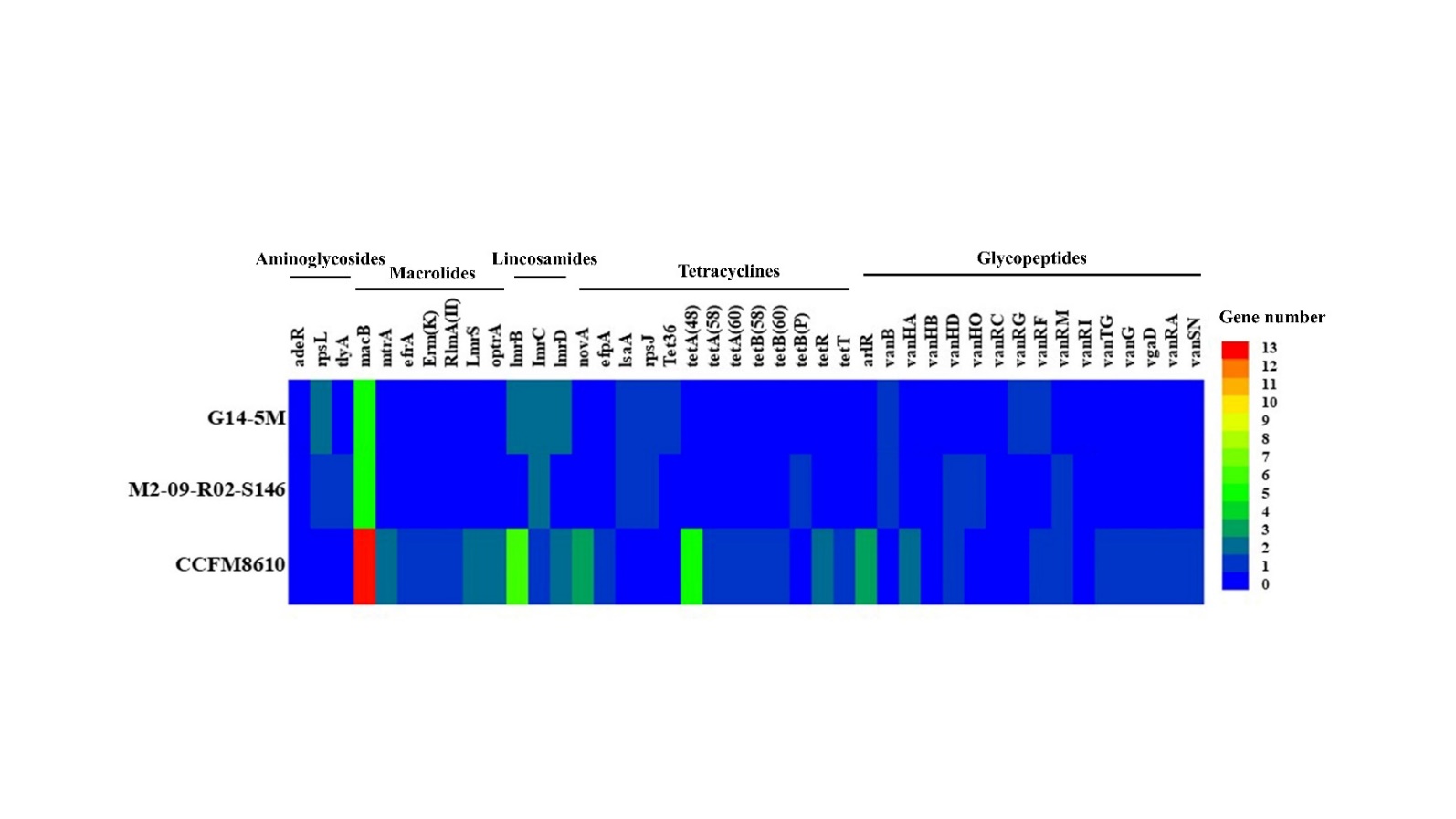
glycopeptides, lincosamides, macrolides, and tetracyclines.

Figure S6: Distribution of resistance genes in *Lactobacillus* strains.

However, the risk of drug resistance genes is directly related to their location. When they are on chromosomes, they are less likely to be transferred and are less harmful, while those on mobile genetic elements (MGEs) are more likely to be transferred with MGEs, allowing other bacteria in the environment to acquire drug resistance genes and are more harmful. Therefore, the study will further analyze the hazard of these drug resistance genes in the context of MGEs.

We compared the prephage of three *Lactobacillus* strains by PHASTER (Table S3). It shown that one complete prephage sequence was identified in each of two *Lactobacillus* strains, while no complete phage region was found in *L. helveticus* M2-09-R02-S146. We further identified the resistance genes on the prephage of these two strains and found that no relevant resistance genes were found on the prephage of *L. crispatus* G14-5M, indicating that the strain has a low risk of transferring resistance genes and a relatively high safety profile.

Table S3: Distribution of the intact prephage regions among *Lactobacillus* strains

| ***Lactobacillus* strain** | **Gene length（kb）** | **The most similar phage** | **GC content（%）** | **Resistance genes** |
| --- | --- | --- | --- | --- |
| G14-5M | 18.7 | PHAGE_Lactob_LfeSau_NC_029068(9) | 34.22 | - |
| M2-09-R02-S146 | - | - | - | - |
| CCFM8610 | 46.4 | PHAGE_Lactob_phig1e_NC_004305(7) | 41.31 | efrA、ImrD |

“-” means none.

To further clarify the resistance of these three *Lactobacillus* strains, the resistance phenotypes were further determined and the strains were classified as resistant or sensitive (MIC < threshold value for sensitive) according to the threshold values specified in the EFSA standard. It was found that some of the resistance phenotypes of these three strains were associated with their resistance genes. All three *Lactobacillus* strains were susceptible to ampicillin and chloramphenicol, which did not have resistance genes, while they all showed resistance to vancomycin, which contained more types and numbers of resistance genes (Table S4).

Table S4: Antibiotic resistance of *Lactobacillus* strains

| **Antibiotic Category** | **Name** | **G14-5M** | **M2-09-R02-S146** | **CCFM8610** |
| --- | --- | --- | --- | --- |
| β-lactam antibiotics | Ampicillin | － | － | － |
| Glycopeptides | Vancomycin | ＋ | ＋ | ＋ |
| Aminoglycosides | Gentamicin | － | － | － |
| Aminoglycosides | Kanamycin | － | － | － |
| Aminoglycosides | Streptomycin | － | － | － |
| Macrolides | Erythromycin | － | － | ＋ |
| Lincosamides | Clindamycin | － | － | － |
| Tetracyclines | Tetracycline | ＋ | － | ＋ |
| Phenylpropanol | Chloromycetin | － | － | － |

“+” indicates resistance, “-” indicates sensitivity.

Overall, all three strains contained at least one CRISPR-Cas system with some resistance to exogenous material invasion. The virulence-related genes carried by the three strains were mainly involved in metabolism, synthesis of extracellular components and adhesion, and there were no significant toxin-producing genes. Although all three strains carried resistance genes, except for *L. plantarum* CCFM8610, the resistance genes of the other two strains were not located on the prephage and had low potential for transfer. In addition, combined with the results of the antibiotic phenotyping experiments, it was found that all of the two strains except *L. plantarum* CCFM8610 showed resistance to vancomycin, while the resistance to other antibiotics was low. *L. crispatus* G14-5M and *L. helveticus* M2-09-R02-S146 were relatively safe. *L. plantarum* CCFM8610 has been widely used and its safety has been proven. Therefore, the three strains can be used in clinical trials.
